# MicroRNA-21 inhibits mitochondria-mediated apoptosis in keloid

**DOI:** 10.18632/oncotarget.21656

**Published:** 2017-10-06

**Authors:** Hao Wu, Jie Wang, Hui Ma, Zhibo Xiao, Xiaoqun Dong

**Affiliations:** ^1^ Department of Plastic and Aesthetic Surgery, The Second Affiliated Hospital of Harbin Medical University, Harbin, China; ^2^ Department of Orthopaedics, The First Affiliated Hospital of General Hospital of The People's Liberation Army, Beijing, China; ^3^ Department of Medicine, Warren Alpert Medical School of Brown University, Providence, Rhode Island, USA

**Keywords:** microRNA-21, mitochondria, apoptosis, keloid, fibroblasts

## Abstract

MicroRNA-21 acts as an oncogene by promoting cell proliferation and migration, whereas inhibiting apoptosis in majority of cancers. MicroRNA-21 is upregulated in human keloid fibroblasts. We hypothesized that microRNA-21 may contribute to pathogenesis of keloid fibroblasts. First, enhanced miR-21 but reduced mitochondrial-mediated apoptosis observed in keloid tissues indicated its importance in keloids development. Second, upregulation of microRNA-21 induced a decrease in the ratio of BAX to BCL-2 and suppressed mitochondrial fission in keloid fibroblasts. Third, by attenuating the decline in cellular mitochondrial membrane potential, overexpression of miR-21 suppressed cytochrome c release to the cytoplasm, followed by a decrease in the activity of intracellular caspase-9 and caspase-3, suggesting that mitochondrial-mediated proapoptotic pathway was impaired. Simultaneously, intracellular reactive oxygen species were decreased, indicating microRNA-21 undermined oxidative stress. This phenotype was reversed by miR-21 inhibition. Therefore, our study demonstrates that inhibition of microRNA-21 induces mitochondrial-mediated apoptosis in keloid fibroblasts, proposing microRNA-21 as a potential therapeutic target in keloid fibroblasts.

## INTRODUCTION

Keloids are more common in Africans and Asians than other races. Keloids can occur at any age, although its age-of-onset mostly ranges from 10 to 30 years old [1–3]. These lesions are defined as a benign fibroproliferative tumor of the skin caused by abnormal healing of cutaneous irritation or injury [4]. Current progress in research provides an ever-deepening insight into the nature of keloid formation. However, the pathogenesis of keloid is only partially understood. So far, a universally optimal therapeutic modality has yet to be established [5–7].

Apoptosis refers to autonomic and programmed cell death in multicellular organisms [8]. Mitochondria have long been termed as ‘powerhouse’ of the cell to synthesize ATP and initiate signal transduction involved in cell death, innate immunity and autophagy [9]. Regulation of mitochondrial apoptosis is mediated by the interaction of *BCL2* family members, such as anti-apoptotic protein BCL2 and pro-apoptotic protein BAX [10]. Proapoptotic insults can activate BH3-only proteins to interact with pro-apoptotic proteins at the mitochondrial outer membrane (MOM). Subsequently, anti-apoptotic proteins can no longer prevent this activation, resulting in mitochondrial outer permeabilization (MOMP) [11–13]. This allows cytochrome c to releases into the cytoplasm and thus to initiate caspases cascade, eventually leading to apoptosis [11, 14].

MiRNAs are small noncoding RNAs which downregulate protein-coding genes [15, 16], while aberrant miRNA expression can cause deleterious phenotypes or diseases [17]. MiRNAs are widely involved in proliferation, differentiation, apoptosis, immune regulation, metabolism, inflammation, tumorigenesis and other pathological processes [18, 19]. MiRNAs may participate in regulating the functions of skin and its appendages as well as the formation of scar [20]. MicroRNA-21 (MiR-21) is abnormally overexpressed in skin wound healing and tumor tissues. Functionally, miR-21 can promote tumor cell proliferation [21–23]. Our previous study has revealed miR-21 to be up-regulated during keloid development [24–26]. It remains unclear whether miR-21 can regulate mitochondria-mediated apoptosis. In the past five years, over one hundred types of antisense oligonucleotide-based therapies have been tested in clinical trials for a variety of diseases [16]. Further exploration for molecular mechanisms will facilitate the development and application of therapeutic modalities.

In this study, we investigated the role of miR-21 in regulating mitochondria-mediated apoptosis pathway in human keloid fibroblasts (KF). This finding may provide theoretical and experimental basis to thoroughly illuminate the mechanism of keloid formation as well as to identify new therapeutic targets.

## RESULTS

### MiR-21 mimic and inhibitor oppositely regulate the expression of miR-21

**Figure 1:**
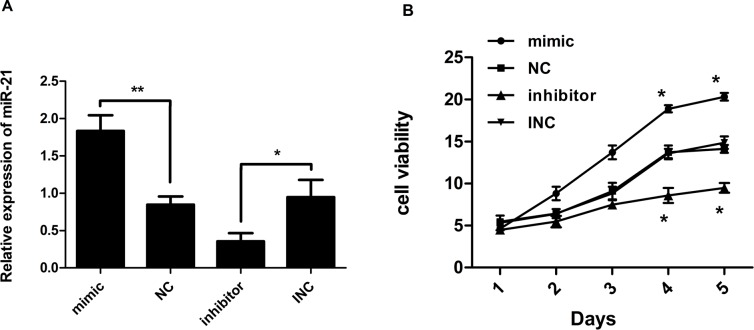
**(A)** Expression ratio of miR-21 relative to U6 small nuclear RNA (n=3). Human keloid fibroblast cells were transfected with miR-21 mimic, negative control (NC), miR-21 inhibitor, and inhibitor negative control (INC), respectively. Relative expression levels of miR-21 were analyzed 24 hours later by RT-PCR. MiR-21 levels had increased vs. decreased significantly in response to mimic vs. inhibitor, respectively, compared with corresponding controls. **(B)** Cell viability measured by CCK8 assay (n=3-5). Compared with corresponding controls, proliferation of keloid fibroblast cells after transfection was significantly reduced in response to miR-21 inhibitor but increased in response to miR-21 mimic; ^**^p< 0.01; ^*^p<0.05. Data are represented as mean ± SD.

We transfected keloid fibroblasts with miR-21 mimic, negative control (NC), miR-21 inhibitors and inhibitor negative control (INC). The transfection efficiency was detected at 24 hours after transfection by real-time quantitative polymerase chain reaction (RT-PCR). MiR-21 level was changed markedly after transfection. Compared with corresponding controls, the difference of miR-21 expression was statistically significant (Figure 1A). It was obviously enhanced in miR-21 mimic transfected cells, whereas decreased after transfection of miR-21 inhibitor.

### Upregulated miR-21 promotes cell proliferation of human KF

The effect of miR-21 on proliferation of keloid fibroblasts was detected by cell counting kit-8 (CCK8) assay. After transfection with miR-21 inhibitor, proliferation of KF cells was significantly reduced compared with INC. In contrast, overexpression of miR-21 led to enhanced proliferation of KF (Figure 1B). This indicated that aberrantly high expression of miR-21 could enhance proliferation of KF.

### Expression of proteins related to mitochondrial apoptosis in keloid tissue

**Figure 2:**
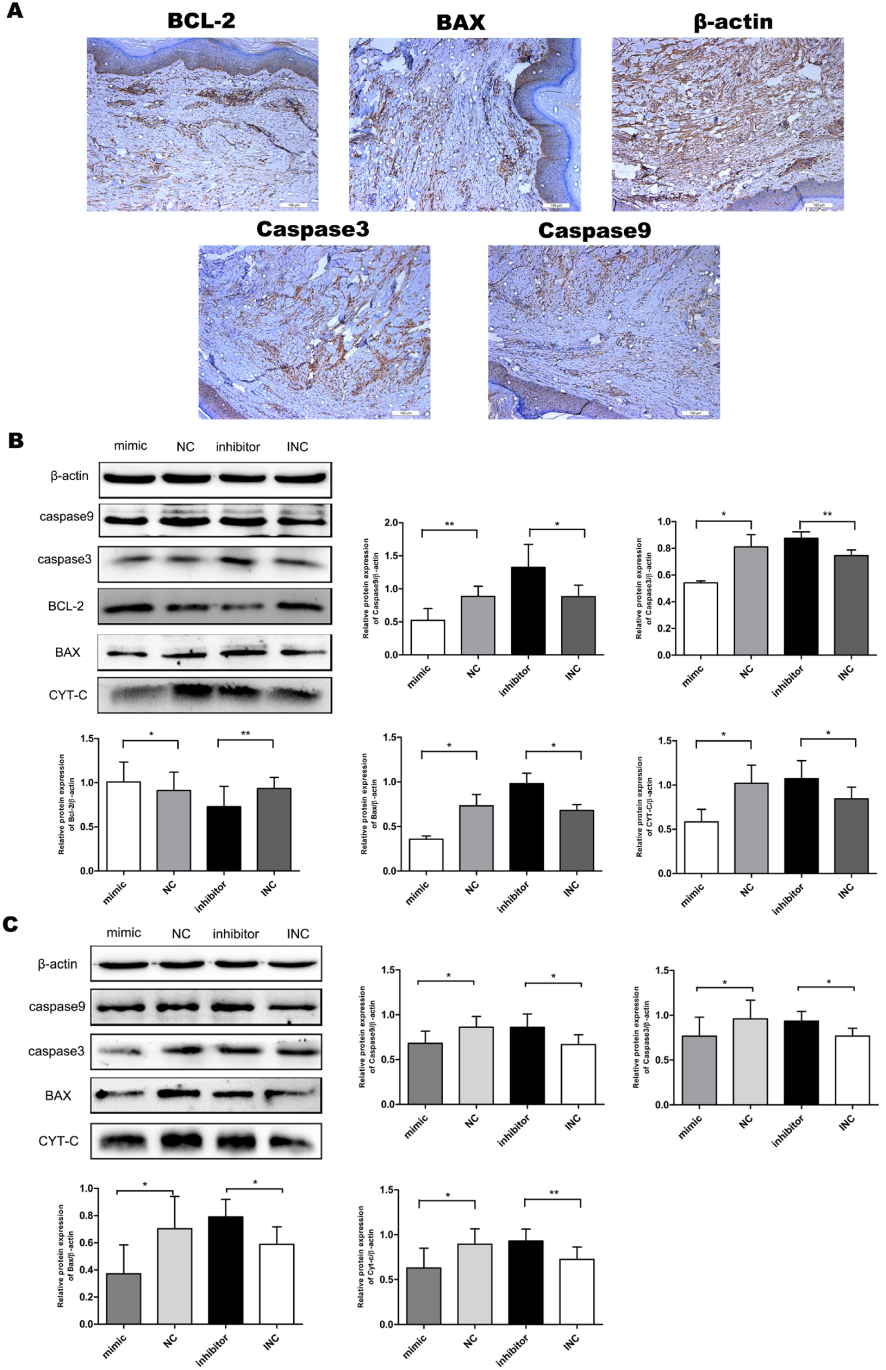
Expression profile of proteins related to mitochondria-mediated apoptosis in keloid fibroblasts **(A)** Expression of apoptotic proteins in keloid tissue by IHC (n=3-6). Bar, 10μm. **(B)** Expression of pro-apoptotic proteins (caspase3, caspase9, CYT-C, BAX) was significantly increased in response to miR-21 inhibitor. In contrast, the expression of anti-apoptotic protein BCL-2 was decreased (n=3-6). **(C)** Inhibition of miR-21 tended to induce an overall shift of apoptotic proteins. To the contrary, mimic miR-21 inhibited apoptosis in normal fibroblasts (n=3-6); ^**^p< 0.01; ^*^p<0.05. Data are represented as mean ± SD.

In order to further explore the mitochondrial apoptosis pathway in keloid tissue, immunohistochemistry (IHC) assay was applied to detect the expression of its components in KF tissue derived from patients (Figure 2A). Important apoptotic proteins in mitochondria (caspase3, caspase9, Bcl-2, Bax) have prominent subsidence in keloid tissue. This finding suggests that mitochondrial apoptotic pathway plays a crucial role in keloid tissue.

### MiR-21 regulated expression of components for mitochondrial apoptosis pathway

Western blot assay was used to measure the expression of pro- and anti-apoptotic proteins in KF (Figure 2B). Compared with INC group, the expression of pro-apoptotic proteins (caspase3, caspase9, CYT-C, BAX) in mitochondria was significantly increased in response to miR-21 inhibitor. In contrast, the expression of anti-apoptotic protein BCL-2 was decreased. Thus, we demonstrated an inhibitory effect of miR-21 on mitochondrial apoptosis in KF. This naturally led us to explore whether overexpression of miR-21 in normal fibroblasts could block apoptosis. We transfected normal fibroblasts with miR-21 mimic, NC, miR-21 inhibitors and INC. In normal fibroblasts, miR-21 had similar effect on mitochondrial apoptosis as shown in Figure 2C. These data indicated that miR-21 could contribute to anti-apoptosis in KF.

### MiR-21 modified mitochondrial fission and membrane potential (MMP) in KF

**Figure 3:**
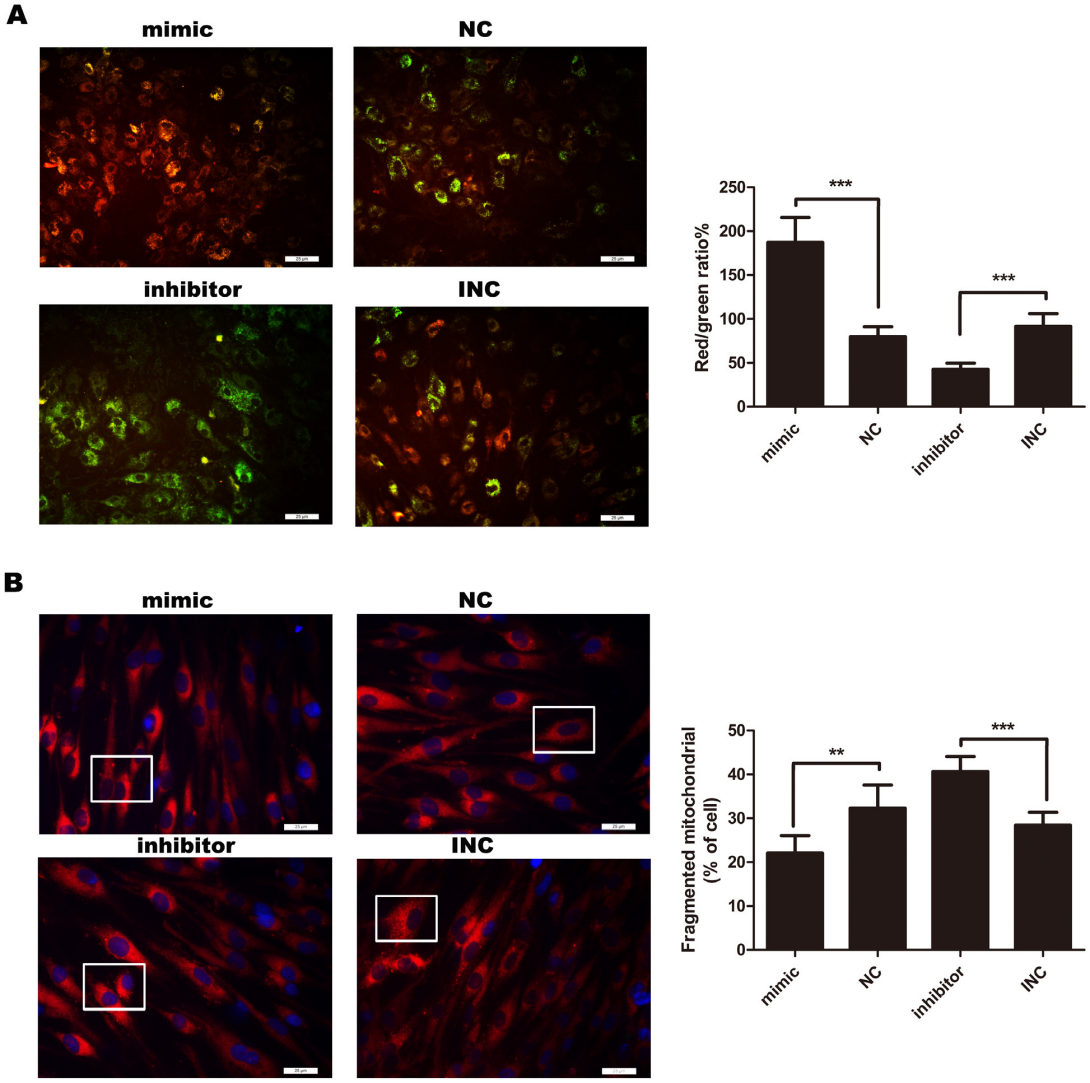
**(A)** Measurement of mitochondrial membrane potential (MMP) (n=4-6). After 12 hours, MMP was detected by JC-10 assay. MMP was decreased in response to inhibition of miR-21, compared with INC. To the contrary, mimic miR-21 inhibited apoptosis by attenuating the decline of MMP. Percentages of J-aggregates (red) and J-monomers (green) were shown in the histogram. Green J-monomers were accumulated in the mitochondria with a low potential; by contrast, red J-aggregates were accumulated in the mitochondria with a high potential. Bar, 25μm. **(B)** Mitochondria were stained by Mito Tracker^®^ Deep Red FM (left panel). Bar, 25μm. Those cells with fragmented mitochondria were counted (right panel) (n=6); ^***^p<0.001; ^**^p< 0.01. Data are represented as mean ± SD.

Mitochondrial fission can mediate apoptosis by leading to a decrease in MMP, which is an early sign of mitochondrial-mediated apoptosis. In our study, MMP was detected by JC-10 assay. Overexpression of miR-21 was accompanied by a decrease in mitochondrial fission (Figure 3B) whereas increased MMP (Figure 3A). As expected, inhibition of miR-21 increased mitochondrial fission (Figure 3B), followed by a marked decrease in MMP (Figure 3A, p<0.001). This indicated that miR-21 could inhibit early mitochondrial-mediated apoptosis pathway.

### MiR-21 inhibits apoptosis in KF

**Figure 4:**
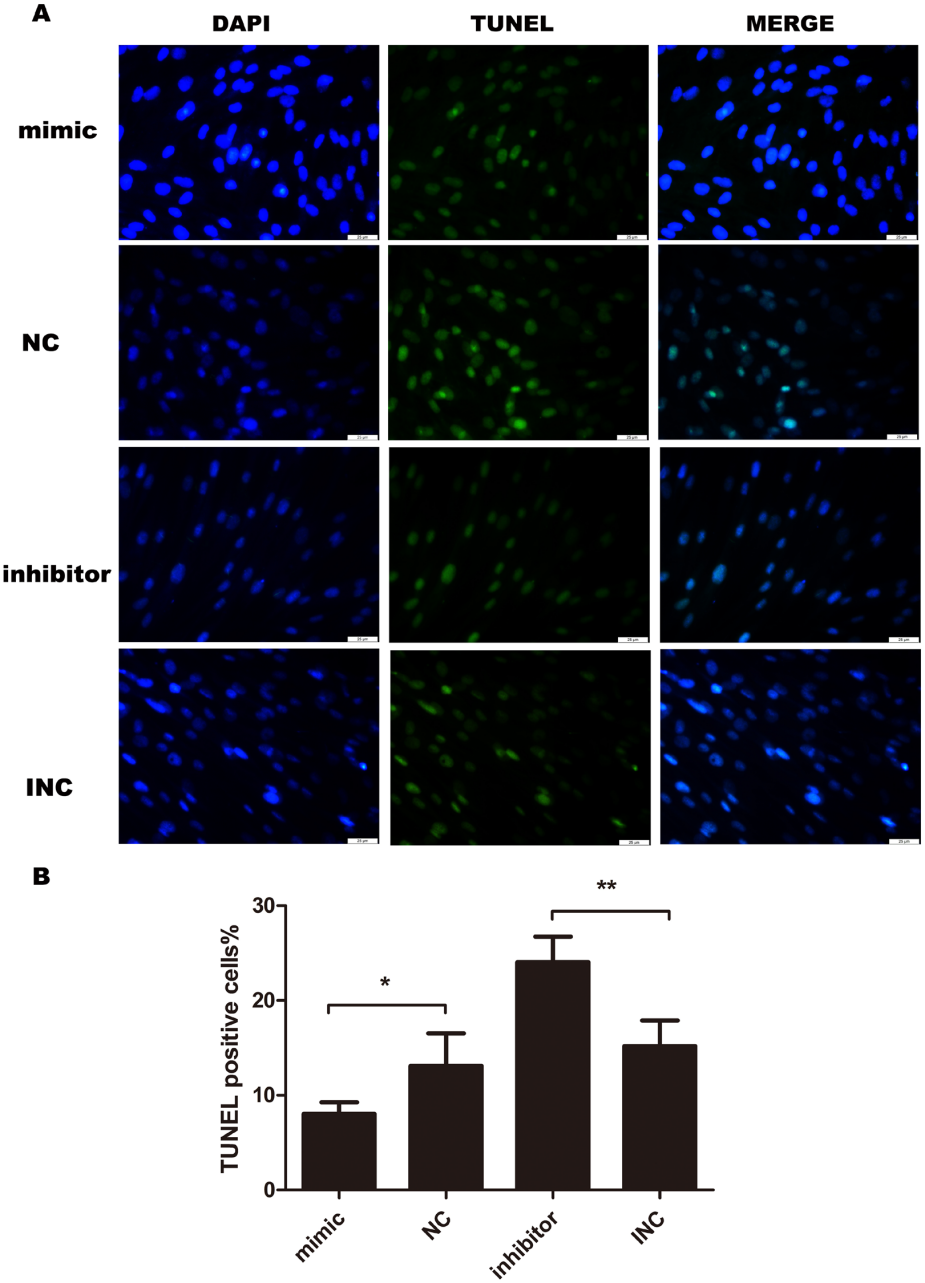
Cell apoptosis evaluated by TUNEL assay after transfection for 24 hours (n=4-5) Transient transfection of miR-21 inhibitor induces apoptosis in keloid fibroblasts, opposite to the effect of miR-21 mimic. Bar, 25μm; ^**^p<0.01; ^*^p<0.05. Data are represented as mean ± SD.

Furthermore, we performed immunofluorescence to analyze the effect of miR-21 on apoptosis by conducting TUNEL assay. Overexpression of miR-21 attenuated terminal deoxyribonucleotidyl transferase-mediated dUTP nick end labeling (TUNEL) positive cells. While, inhibition of miR-21 increased TUNEL positive cells (Figure 4). The results suggested that miR-21 inhibited cell apoptosis while inhibition of miR-21 induced apoptosis in KF

### MiR-21 induces resistance to oxidative stress in KF

**Figure 5:**
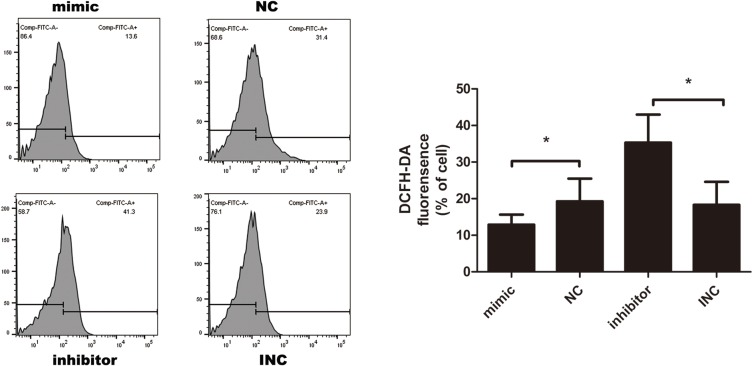
Intracellular reactive oxygen species (ROS) measured by DCFH-DA probe after transfection for 12 hours (n=3-6) Transient transfection of miR-21 inhibitor elevated ROS level, opposite to the effect of miR-21 mimic; ^*^p<0.05. Data are represented as means ± SD.

As reactive oxygen species (ROS) produced by oxidative stress can act as signal molecules to mediate apoptosis, we further detected intracellular ROS level by DCFH-DA probe. As shown in the Figure 5, green fluorescent DCFH-DA probe was loaded into cells. According to the principle of DCFH-DA staining, DCFH-DA is diffused into cells that generate ROS, the stronger the green fluorescence, the higher the ROS level. Upregulation of miR-21 resulted in a decrease in ROS while inhibition of miR-21 increased ROS level, indicating that miR-21 might induce resistance to oxidative stress.

## DISCUSSION

Although many studies have been performed to explore the etiology of keloids, the molecular mechanism have not been well defined. In recent years, genetic and epigenetic factors responsible for human excessive scarring have been identified by genome and epigenome sequencing [5]. However, only a few genes responsible for keloids have been found, including several HLA alleles (HLA-DRB1^*^15, HLA-DQA1^*^0104, DQ-B1^*^0501, and DQB1^*^0503), 25 dysregulated genes associated with apoptosis, mitogen-activated protein kinase (MAPK), transforming growth factor (TGF)-β, interleukin (IL)-6, and plasminogen activator inhibitor (PAI)-1 as well as 32 dysregulated miRNAs [24, 27]. Some studies suggest that genomic alterations in death ligands may affect cell death pathways in defining sensitivity to specific therapies [28, 29]. This finding highlights the role of genomic alterations in modifying diseases development and clinical efficacy. The novelty of this study is to provide assertive evidence for further understanding of keloids development and treatment by inhibiting miR-21 induced anti-apoptosis in KF.

Firstly, miRNA, 19-22nt long noncoding RNA, is a large family that regulates gene expression [20]. In the genome, miRNA is distributed in the noncoding region of DNA. It could consist in introns of protein coding genes or intron and exon regions of noncoding genes. Therefore, miRNAs play a crucial role in transcriptional regulation of host genes. These miRNAs are either isolated or clustered together [20–21, 30–32]. One miRNA can regulate multiple genes, and one gene can be regulated by multiple miRNAs. In a particular organization, a specific miRNA regulates a particular set of mRNAs. MiRNAs participate in developmental biology, and distribution of cells and tissues [33–35]. Thus, miRNAs have significant therapeutic potential because they regulate multiple gene targets in a particular signaling pathway, or plenty of targets across several independent pathways [17, 36].

Secondly, miR-21, first identified by Lagos-Quintana in 2001 [37], is encoded by a single gene localized on 17q23.2, the tonoplast protein coding region and the tenth intron of the VMP1 gene [38]. Most studies confirm that miR-21 is overexpressed in various tumor samples and could stimulate tumor cell growth and proliferation [39–48]. MiR-21 is one of the 23 upregulated miRNAs in keloid tissues [24]. We have elucidated that miR-21 regulates PTEN/AKT and TGF-β1 signaling pathways to promote KF proliferation and transdifferentiation [25, 26]. This indicates miR-21 as a potential therapeutic target for keloids. In this study, we found miR-21 could inhibit mitochondrial-mediated apoptosis pathway in KF.

Thirdly, apoptosis (programmed cell death) is considered vital for embryonic development, normal cell turnover, hormone-dependent atrophy, proper development and functioning of the immune system, and chemical-induced cell death [49]. Mitochondria are important integrators and critically involved in apoptosis. The mitochondria are an organelle to produce active oxygen (ROS) which is an important component of mitochondrial respiratory chain and oxidative phosphorylation in cells. ROS is the product of aerobic metabolism *in vivo*, mainly derived from mitochondria. Notably, 95%-98% of molecular oxygen in the organism is consumed by mitochondria to produce water, and 2%-5% is used to produce super oxide and hydrogen peroxide via the electron transport chain [50]. Any factor that reduces the efficiency of the electron transport process can increase the production of ROS. ROS produced by oxidative stress can act as signal molecules to mediate apoptosis [51]. In our study, miR-21 mimic transfection inhibited ROS triggered mitochondrial apoptosis in KF. At the same time, the release of cytochrome c is a major step in endogenous apoptosis pathway. The release of cytochrome c to cytoplasm leads to the formation of cytochrome c/apoptosis protease activating factor apoptosome. This complex promotes self-activation of caspase-9 precursor and accelerates the activation of caspase-3 precursor by caspase-9, eventually resulting in apoptosis. Similarly, cytochrome c can cause changes in membrane symmetry, causing nuclear shrinkage, DNA fragmentation and cell death [52]. The *BCL2* family is the earliest reported apoptosis related gene. BCL2 is a key factor in many pathological and physiological processes through inhibiting apoptosis [53]. Both BAX and BCL-x are members of *BCL2* family, which can form a homodimer. The ratio between these two factors determines the fate of cells [54]. If the concentration of BAX increases, the scale of BAX and BCL2 will be tipped to promote apoptosis [55].

In this study, we evaluated the effects of both miR-21 mimic and miR-21 inhibitor on mitochondrial apoptotic pathway in KF. In order to prove that miR-21 could block mitochondrial apoptosis pathway, miR-21 mimic was transfected into normal skin fibroblasts. Interestingly, pro-apoptotic proteins were downregulated. In KF, upregulation of miR-21 attenuates mitochondrial-mediated apoptosis by reducing the ratio of pro-apoptotic protein BAX to anti-apoptotic protein BCL2 (BAX/BCL2), accompanied with downregulation of cytochrome c, caspase 9, and caspase 3. While inhibition of miR-21 leads to increased BAX/BCL2 ratio as well as mitochondrial fission followed by decreased MMP, thus increased cytochrome c, caspase 9, and caspase 3. This overall shift is critical for synergistic induction of mitochondrial-mediated apoptosis. In addition, TUNEL assay has shown that inhibition of miR-21 rescues apoptosis.

Most of targets for miR-21 have been identified as tumor suppressors, such as PDCD4 (Programmed cell death 4), PTEN, RECK, TP63 and FASLG [56]. For example, miR-21 can be transcriptionally activated by NF-κB and downregulate phosphatases PDCD4 and PTEN. BCL2 is a anti-apoptotic factor, regulating caspase activity by sequestering cytochrome c through inhibiting mitochondria-permeabilzing protein BAX. In KF, miR-21 may directly downregulate BAX and upregulate BCL2, leading to an increased ratio of BCL2/BAX, and eventually inhibiting apoptosis. In addition, other targets of miR-21 may significantly contribute to KF development. This needs to be experimentally validated.

So far, canonical miRNAs including miR-21 and others like miR-34, miR-155, and miR-200b have been developed or are in development into therapeutic drugs to treat specific patients with chronic diseases, especially tumors [57–61]. Further exploration of miR-21 in common diseases such as keloids will facilitate the development and application of therapeutic modalities.

In conclusion, our study demonstrates that miR-21 precludes mitochondrial-mediated apoptosis in KF, which highlights miR-21 as a potential target for prevention and treatment of keloids. Undoubtedly, there are many other factors and pathways can influence this process. Multiple effects of miR-21 and other elements on the pathogenesis of KF require further investigation.

## MATERIALS AND METHODS

### Patient samples

Keloid and normal skin tissue samples were obtained from 13 patients (3 males, 10 females) who underwent operations from 2016 to 2017 at the Second Affiliated Hospital of Harbin Medical University. Informed consent was obtained from each patient recruited, and the study was approved by the hospital ethics committee. Keloid cells were obtained at surgical release from seven patients aged 20 to 40 years who had a nonpeduncular keloid on the ear lobe, main trunk, and upper arm of at least 1-year evolution, with clinical activity such as growth, pain, pruritus, and hyperemia. None of them had been treated previously.

### Cell culture

Keloid tissues and normal skin tissues were washed with phosphate-buffered saline (PBS) separately, cut into small pieces. Finally, they were adhered and cultured on the bottom of tissue culture flasks. After 3-5 d of culture, the KF grew out of tissues. For the normal skin fibroblasts, they grew out after 5-7 d of culture. Primary KF and normal fibroblasts were collected and then cultured in DMEM (Corning, USA) supplemented with 10% fetal bovine serum (Corning, USA) at 37°C, under a 5.0%CO_2_ atmosphere. Primary KF and NF used in this study were retained in the third to seventh passage.

### Transient transfection

The miRNA-21 mimic, negative control, miRNA-21 inhibitor, and inhibitor negative control were purchased from Genepharma Biotechnology (Shanghai, China) and the sequences were shown in Table 1. Cells were transfected with Lipofectamine 2000 (Invitrogen, USA) at a final concentration of 100 nM following the manufacturer's instructions. At the indicated time for various assays cells were collected.

**Table 1:** Sequence of miR-21 oligonucleotides for transient transfection

Name	Sequence (5′-3′)
miR-21 mimic	UAGCUUAUCAGACUGAUGUUGA
NC	UUCUCCGAACGUGUCACGUTT
miR-21 inhibitor	UCAACAUCAGUCUGAUAAGCUA
INC	CAGUACUUUUGUGUAGUACAA

### RNA extraction and real-time PCR

Trizol Reagent (Invitrogen, USA) was used to extract total RNA from the cells. Hairpin-it miRNAs quantitative and U6 snRNA normalization RT-PCR Quantitation Kit (GenePharma, China) was used to reverse transcription RNA into cDNA and perform RT-PCR for miR-21 and U6. According to the manufacturer's protocol, RNA samples were transcribed into cDNA. Equal amounts of cDNA samples were used for real-time quantitative PCR detection and U6 was used as an internal standard. The primer sequences used were as follows: miR-21, 5′-GGCAGCCTAGCTTATCAGACT-3′ (forward); 5′-GTGCAGGGTCCGAGGTATTC-3′ (reverse); and U6, 5′-CTCGCTTCGGCAGCACA-3′ (forward); 5′-AACGCTTCACGAATTTGCGT-3′ (reverse). RNA expression was measured by real-time PCR in ABI Prism 7500 Sequence Detection System (Applied Biosystems, USA). The PCR conditions used were: miR-21, 3 minutes at 95°C, 40 cycles of 12 seconds at 95°C and 40 seconds at 62°C. Melt curve analysis was performed to determine the specificity of the reaction. Each sample was tested in triplicate. Relative expression level changes were using the 2^−ΔΔCt^ method.

### Cell proliferation assay

CCK8 assay (Dojindo, China) was performed to assess the role of miRNA-21 in keloid fibroblasts. Cells cultured in DMEM (10% FBS) were seeded into 96-well plates and transfected with miRNA-21 mimics or inhibitor. At 1, 2, 3, 4, 5 days after transfection, 10μl CCK8 in 90μl DMEM was added into each well. After incubated for 2 hours, the absorbance was measured at 450nm using a microplate reader (BIO-RAD, iMarker, USA).

### Protein isolation and western blot

Firstly, separation of total protein components, KF and normal skin fibroblasts (after 72 hours' transfection) were collected, washed twice with ice-cold phosphate-buffered saline, and lysed using cell lysis buffer [20 mM Tris (pH 7.5), 150 mM sodium chloride, 1% Triton X- 100, 2.5 mM sodium pyrophos-phate, 1 mM ethylenediaminetetraacetic acid, 1% sodium carbonate, 0.5 μg/ml leupeptin, and1 mM phenylmethanesulfonyl fluoride]. Scraper collected lysate and then centrifuged at 12,000 rpm at 4°C for 15 minutes. Total protein samples (20 μg) were loaded onto a 12% or 15% of sodium dodecyl sulfate polyacrylamide gel for electrophoresis, and transferred onto polyvinyl difluoride transfer membranes (Roche, USA) at 0.8 mA/ cm^2^ for 60 minutes. Membranes were blocked at 37°C for 1 hour with blocking solution (1% bovine serum albumin in phosphate-buffered saline plus 0.05% Tween-20). The samples were incubated overnight at 4°C with primary anti-bodies for β-actin (1:8000), bax (1:1000), bcl-2 (1:5000), cyt-c (1:4000), caspase 9 (1:2000) and caspase 3 (1:2000) (abcam, USA) at a compatible dilution in blocking solution. Wash three steps in Tris -buffered saline Tween-20 for 10 minutes each, membranes were incubated for 2 hours at room temperature with an anti-rabbit secondary antibody (abcam, USA) at a dilution of 1:8000 in blocking solution. After washing three times for 10 minutes each time in Tris- buffered saline Tween-20, signals were visualized using an enhanced chemiluminescence kit (Beyotime BioTech, China).

### Immunohistochemical assay

Following formalin fixation and paraffin-embedding, the 4-μm thick keloid tissue sections were incubated with primary anti-bodies for β-actin (1:1000), bax (1:400), bcl-2 (1:500), caspase 9 (1:250) and caspase 3 (1:500) (abcam, USA) overnight at 4°C, washed thrice with PBS for 10 min each, and then incubated with the secondary antibody (1:1000) (abcam, USA) for 1h at 37°C. Finally, the sections were stained with 3,3′-diaminobenzidine and then counterstained with hematoxylin. Pictures were taken with Leica microscope (Leica, Germany).

### Mitochondrial membrane potential (MMP)

MMP was evaluated by mitochondrial membrane potential (JC-10 probe) detection kit (Solarbio, China) as described by the manufacturer. After transfection for 12 hours, the cells were incubated for 20 min at 37°C in a humidified atmosphere in the dark. Positive control cells added with cccp (10μM) and the mitochondrial membrane potential significant changes. Following incubation, JC-10 buffer solution washed each well for 100μl/ml for twice, each time was 5 min. Analysed by inverted fluorescence microscope (Leica, Germany).

### Mitochondrial fission

Mitochondrial fission was detected by Mito Tracker^®^ Deep Red FM (Yeasen, China) as described by the manufacturer. After transfection for 12 hours, the cells were incubated for 20 min at 37°C in a humidified atmosphere in the dark. Pictures were taken with Leica microscope (Leica, Germany).

### Cell apoptosis assay

TUNEL assay was performed to measure cell apoptosis using *in situ* cell death detection kit, fluorescein (Roche, USA). After transfection for 24 hours, cells were fixed with 2% formaldehyde at room temperature for 1 hour and permeabilized with 0.1% Triton X-100 (Solarbio, China) for 2 minutes on ice. After three wash steps with PBS, the cells were incubated for 1 hour at 37°C in a humidified atmosphere in the dark with 50μl TUNEL reaction mixture according to instructions of the manufacturer. Analysed by inverted fluorescence microscope (Leica, Germany).

### Intracellular ROS detection assay

Intracellular ROS was detected using a DCFH-DA fluorescence probe (Beyotime BioTech, China). After transfection for 12 hours, the cells were treated with DCFH-DA (10μM) and incubated for 30 min at 37°C in a humidified atmosphere in the dark. Analysed by flow cytometry (BD FACS cantoII, USA).

### Statistical analysis

For statistical analyses, all data are expressed as the means ± SD. The difference between two groups were compared using the Student's t-test. Values of p<0.05 were considered statistically significant. GraphPad Prism 5.0 software was used for the statistical analysis.

## Abbreviations

miR-21: microRNA-21
KF: keloid fibroblasts
MMP: mitochondrial membrane potential
ROS: reactive oxygen species
MOM: mitochondrial outer membrane
MOMP: mitochondrial outer permeabilization
NC: negative control
INC: inhibitor negative control
RT-PCR: real-time quantitative polymerase chain reaction
CCK8: cell counting kit-8
IHC: immunohistochemistry
TUNEL: terminal deoxyribonucleotidyl transferase-mediated dUTP nick end labeling
MAPK: mitogen-activated protein kinase
TGF: transforming growth factor
IL: interleukin
PAI: plasminogen activator inhibitor
PBS: phosphate-buffered saline

## References

[1] Fitzpatrick TB, Eisen AZ, Wolff K, Freedberg IM, Austen KF (1979). Neoplasms, pseudoneoplasms and hyperplasias of supporting tissue origin.. Dermatology in General Medicine.

[2] Murray JC, Pollack SV, Pinnell SR (1981). Kelolds: a review. J Am Acad Dermatol.

[3] Rook A, Wilkinson DS, Ebling FJ (1972). Textbook of Dermatology.

[4] Tredget EE, Nedelec B, Scott PG, Ghahary A (1997). Hypertrophic scars, keloids, and contractures. The cellular and molecular basis for therapy. Surg Clin North Am.

[5] Trace AP, Enos CW, Mantel A, Harvey VM (2016). Keloids and hypertrophic scars: a spectrum of clinical challenges. Am J Clin Dermatol.

[6] Van den Broek LJ, Limandjaja GC, Niessen FB, Gibbs S (2014). Human hypertrophic and keloid scar models: principles, limitations and future challenges from a tissue engineering perspective. Exp Dermatol.

[7] Bock O, Schmid-Ott G, Malewski P, Mrowietz U (2006). Quality of life of patients with keloid and hypertrophic scarring. Arch Dermatol Res.

[8] Kerr JF, Wyllie AH, Currie AR (1972). Apoptosis: a basic biologic phenomenon with wide-ranging implications in tissue kinetics. Br J Cancer.

[9] Tait SW, Green DR (2012). Mitochondria and cell signalling. J Cell Sci.

[10] Maes ME, Schlamp CL, Nickells RW (2017). BAX to basics: how the BCL2 gene family controls the death of retinal ganglion cells. Prog Retin Eye Res.

[11] Tait SW, Green DR (2010). Mitochondria and cell death: outer membrane permeabilization and beyond. Nat Rev Mol Cell Biol.

[12] Chipuk JE, Moldoveanu T, Llambi F, Parsons MJ, Green DR (2010). The BCL-2 family reunion. Mol Cell.

[13] Tait SW, Parsons MJ, Llambi F, Bouchier-Hayes L, Connell S, Muñoz-Pinedo C, Green DR (2010). Resistance to caspase-independent cell death requires persistence of intact mitochondria. Dev Cell.

[14] Taylor RC, Cullen SP, Martin SJ (2008). Apoptosis: controlled demolition at the cellular level. Nat Rev Mol Cell Biol.

[15] Adams BD, Parsons C, Walker L, Zhang WC, Slack FJ (2017). Targeting noncoding RNAs in disease. J Clin Invest.

[16] Friedman RC, Farh KK, Burge CB, Bartel DP (2009). Most mammalian mRNAs are conserved targets of microRNAs. Genome Res.

[17] Adams BD, Kasinski AL, Slack FJ (2014). Aberrant regulation and function of microRNAs in cancer. Curr Biol.

[18] Poormasjedi-Meibod MS, Salimi Elizei S, Leung V, Baradar Jalili R, Ko F, Ghahary A (2016). Kynurenine modulates MMP-1 and type-I collagen expression via aryl hydrocarbon receptor activation in dermal fibroblasts. J Cell Physiol.

[19] Stadler BM, Ruohola-Baker H (2008). Small RNAs: keeping stem cells in line. Cell.

[20] Wojtas B, Ferraz C, Stokowy T, Hauptmann S, Lange D, Dralle H, Musholt T, Jarzab B, Paschke R, Eszlinger M (2014). Differential miRNA expression defines migration and reduced apoptosis in follicular thyroid carcinomas. Mol Cell Endocrinol.

[21] Bronevetsky Y, Ansel KM (2013). Regulation of miRNA biogenesis and turnover in the immune system. Immunol Rev.

[22] Fan X, Chen J, Shi D, Jia J, He J, Li L, Lei T, Chen X (2016). The role and mechanisms of action of SIRT6 in the suppression of postoperative epidural scar formation. Int J Mol Med.

[23] Armour A, Scott PG, Tredget EE (2007). Cellular and molecular pathology of HTS: basis for treatment. Wound Repair Regen.

[24] Liu Y, Yang D, Xiao Z, Zhang M (2012). miRNA expression profiles in keloid tissue and corresponding normal skin tissue. Aesthetic Plast Surg.

[25] Liu Y, Wang X, Yang D, Xiao Z, Chen X (2014). MicroRNA-21 affects proliferation and apoptosis by regulating expression of PTEN in human keloid fibroblasts. Plast Reconstr Surg.

[26] Liu Y, Li Y, Li N, Teng W, Wang M, Zhang Y, Xiao Z (2016). TGF-β1 promotes scar fibroblasts proliferation and transdifferentiation via upregulating MicroRNA-21. Sci Rep.

[27] Shih B, Bayat A (2010). Genetics of keloid scarring. Arch Dermatol Res.

[28] Sun Q, Zheng X, Zhang L, Yu J (2011). Smac modulates chemosensitivity in head and neck cancer cells through the mitochondrial apoptotic pathway. Clin Cancer Res.

[29] Raulf N, El-Attar R, Kulms D, Lecis D, Delia D, Walczak H, Papenfuss K, Odell E, Tavassoli M (2014). Differential response of head and neck cancer cell lines to TRAIL or Smac mimetics is associated with the cellular levels and activity of caspase-8 and caspase-10. Br J Cancer.

[30] Daniels JT, Schultz GS, Blalock TD, Garrett Q, Grotendorst GR, Dean NM, Khaw PT (2003). Mediation of transforming growth factor-beta(1)-stimulated matrix contraction by fibroblasts: a role for connective tissue growth factor in contractile scarring. Am J Pathol.

[31] Rao R, Nagarkatti P, Nagarkatti M (2015). Role of miRNA in the regulation of inflammatory genes in staphylococcal enterotoxin B-induced acute inflammatory lung injury and mortality. Toxicol Sci.

[32] Ashburner M, Ball CA, Blake JA, Botstein D, Butler H, Cherry JM, Davis AP, Dolinski K, Dwight SS, Eppig JT, Harris MA, Hill DP, Issel-Tarver L (2000). Gene ontology: tool for the unification of biology. The Gene Ontology Consortium. Nat Genet.

[33] Li Y, Zhuang L, Wang Y, Hu Y, Wu Y, Wang D, Xu J (2013). Connect the dots: a systems level approach for analyzing the miRNA-mediated cell death network. Autophagy.

[34] Cai T, Liu ZH, Wang ZX, Zhao M, Ju HL, Li JQ (2013). miRNA in regulation of skin and hair follicle development. Yi Chuan.

[35] Roy S, Sen CK (2012). miRNA in wound inflammation and angiogenesis. Microcirculation.

[36] Esquela-Kerscher A, Slack FJ (2006). Oncomirs-microRNAs with a role in cancer. Nat Rev Cancer.

[37] Lagos-Quintana M, Rauhut R, Lendeckel W, Tuschl T (2001). Identification of novel genes coding for small expressed RNAs. Science.

[38] Xu XM, Qian JC, Deng ZL, Cai Z, Tang T, Wang P, Zhang KH, Cai JP (2012). Expression of miR-21, miR-31, miR-96 and miR-135b is correlated with the clinical parameters of colorectal cancer. Oncol Lett.

[39] Chang KH, Miller N, Kheirelseid EA, Ingoldsby H, Hennessy E, Curran CE, Curran S, Smith MJ, Regan M, McAnena OJ, Kerin MJ (2011). MicroRNA-21 and PDCD4 expression in colorectal cancer. Eur J Surg Oncol.

[40] Kanaan Z, Rai SN, Eichenberger MR, Roberts H, Keskey B, Pan J, Galandiuk S (2012). Plasma miR-21: a potential diagnostic marker of colorectal cancer. Ann Surg.

[41] Liu ZL, Wang H, Liu J, Wang ZX (2013). MicroRNA-21 (miR-21) expression promotes growth, metastasis, and chemo- or radioresistance in non-small cell lung cancer cells by targeting PTEN. Mol Cell Biochem.

[42] Sheedy FJ, Palsson-McDermott E, Hennessy EJ, Martin C, O'Leary JJ, Ruan Q, Johnson DS, Chen Y, O'Neill LA (2010). Negative regulation of TLR4 via targeting of the proinflammatory tumor suppressor PDCD4 by the microRNA miR-21. Nat Immunol.

[43] Gao W, Yu Y, Cao H, Shen H, Li X, Pan S, Shu Y (2010). Deregulated expression of miR-21, miR-143 and miR-181a in non small cell lung cancer is related to clinicopathologic characteristics or patient prognosis. Biomed Pharmacother.

[44] Yang M, Shen H, Qiu C, Ni Y, Wang L, Dong W, Liao Y, Du J (2013). High expression of miR-21 and miR-155 predicts recurrence and unfavourable survival in non-small cell lung cancer. Eur J Cancer.

[45] Li H, Cheng J, Mao Y, Jiang M, Fan X (2015). miR-21 inhibits the effects of cyclooxygenase-2 inhibitor NS398 on apoptosis and invasion in gastric cancer cells. Onco Targets Ther.

[46] Li L, Li C, Wang S, Wang Z, Jiang J, Wang W, Li X, Chen J, Liu K, Li C, Zhu G (2016). Exosomes derived from hypoxic oral squamous cell carcinoma cells deliver miR-21 to normoxic cells to elicit a prometastatic phenotype. Cancer Res.

[47] Zhang JT, Cai QY, Ji SS, Zhang HX, Wang YH, Yan HT, Yang XJ (2016). Decreased miR-143 and increased miR-21 placental expression levels are associated with macrosomia. Mol Med Rep.

[48] Mourelatos C, Nikolaropoulos S, Fousteris M, Pairas G, Argyraki M, Kareli D, Dafa E, Mourelatos D, Lialiaris T (2012). Synergistic cytogenetic and antineoplastic effects by the combined action of esteric steroidal derivatives of nitrogen mustards. Genet Test Mol Biomarkers.

[49] Danial NN, Korsmeyer SJ (2004). Cell death: critical control points. Cell.

[50] Jacobson J, Duchen MR (2001). Mitochondrial oxidative stress and cell death in astrocytes--requirement for stored Ca2+ and sustained opening of the permeability transition pore. J Cell Sci.

[51] Dallak MM, Mikhailidis DP, Haidara MA, Bin-Jaliah IM, Tork OM, Rateb MA, Yassin HZ, Al-Refaie ZA, Ibrahim IM, Elawa SM, Rashed LA, Afifi NA (2008). Oxidative stress as a common mediator for apoptosis induced-cardiac damagein diabetic rats. Open Cardiovasc Med J.

[52] Chawla-Sarkar M, Leaman DW, Borden EC (2001). Preferential induction of apoptosis by interferon (IFN)-beta compared with IFN-alpha2: correlation with TRAIL/Apo2L induction in melanoma cell lines. Clin Cancer Res.

[53] Bissonnette RP, Echeverri F, Mahboubi A, Green DR (1992). Apoptotic cell death induced by c-myc is inhibited by bcl-2. Nature.

[54] Schlaifer D, Krajewski S, Galoin S, Rigal-Huguet F, Laurent G, Massip P, Pris J, Delsol G, Reed JC, Brousse P (1996). Immunodetection of apoptosis-regulating proteins in lymphomas from patientswith and without human immunodeficiency virus infection. Am J Pathol.

[55] Oltvai ZN, Milliman CL, Korsmeyer SJ (1993). Bcl-2 heterodimerizes in vivo with a conserved homolog, Bax, that acceleratesprogrammed cell death. Cell.

[56] Buscaglia LE, Li Y (2011). Apoptosis and the target genes of microRNA-21. Chin J Cancer.

[57] Ge Y, Zhang L, Nikolova M, Reva B, Fuchs E (2016). Strand-specific *in vivo* screen of cancer-associated miRNAs unveils a role for miR-21(^*^) in SCC progression. Nat Cell Biol.

[58] Adams BD, Wali VB, Cheng CJ, Inukai S, Booth CJ, Agarwal S, Rimm DL, Győrffy B, Santarpia L, Pusztai L, Saltzman WM, Slack FJ (2016). miR-34a silences c-SRC to attenuate tumor growth in triple-negative breast cancer. Cancer Res.

[59] Babar IA, Cheng CJ, Booth CJ, Liang X, Weidhaas JB, Saltzman WM, Slack FJ (2012). Nanoparticle-based therapy in an in vivo microRNA-155 (miR-155)-dependent mouse model of lymphoma. Proc Natl Acad Sci U S A.

[60] Yao L, Daniels J, Moshnikova A, Kuznetsov S, Ahmed A, Engelman DM, Reshetnyak YK, Andreev OA (2013). pHLIP peptide targets nanogold particles to tumors. Proc Natl Acad Sci U S A.

[61] Mitra RN, Nichols CA, Guo J, Makkia R, Cooper MJ, Naash MI, Han Z (2016). Nanoparticle-mediated miR200-b delivery for the treatment of diabetic retinopathy. J Control Release.

